# Tribological Characteristic of a Ring Seal with Graphite Filler

**DOI:** 10.3390/ma13020311

**Published:** 2020-01-09

**Authors:** Wojciech Szczypinski-Sala, Janusz Lubas

**Affiliations:** 1 Faculty of Mechanical Engineering, Cracow University of Technology, Jana Pawla II 37, 31-864 Cracow, Poland; 2 The Faculty of Mechanical Engineering and Aeronautics, Rzeszow University of Technology, Powstańców Warszawy 8, 35-959 Rzeszów, Poland

**Keywords:** composite materials, rubber O-ring seal, tribology, friction

## Abstract

This paper presents the outcome of the measurement of the tribological characteristic of O-ring seals in the event of operating in conditions with a lack of lubrication. The measurement was carried out on a seal and rod model. The measurement was carried out during the condition of the round cross-section seal sliding on the surface of the piston rod. We analyzed how the friction force during rod movement, which resulted from the cooperation of the sliding nod and the rod, was changing. The experiment was conducted for various rubber materials. The aim of the research was to evaluate the friction reducing capability of graphite in rubbers of commercial sealing parts. Typical materials used for the seal and the materials, which contained the filler in the form of graphite powder, were compared. Synthetic graphite powder with a particle size of 1–2 µm was applied, and nitrile rubber (NBR) and fluoroelastomer (FKM) were compared as typical materials for O-ring seals. In the case of the two tested materials, the addition of graphite powder had an influence on the decrease in the friction force.

## 1. Introduction

Rubber O-ring seals are one of the cheapest and simplest types of seals, and are, therefore, extensively used in mechanical systems [1]. O-ring seals can prevent the leakage of various media. They can operate as a separate seal or be an element of a sealing kit. They operate in different conditions, including scarce lubrication or even the lack of it. From the point of view of their reliability, properties of the material from which they are made are of vital significance [2,3,4,5,6,7,8]. 

During friction in conditions without lubrication, friction force can reach very high values during relative movement. In applications such as ring seals, this is an unfavorable phenomenon. In conditions of reciprocation movement, one can always observe higher friction force at the beginning of the rod stroke. It is especially visible when the elements remain motionless for a long time. Therefore, also in case of the materials used for sealing rings, it is important to evaluate their tribological properties in conditions of friction without lubrication. In such conditions, the surface of the sealing element can quickly become worn and damaged. As a result, at short notice, the element will not fulfill its task. It is vital to understand the phenomenon of friction and wear [9,10]. The possibility of extension of the operation period, as well as prediction of wear and probability of failure, are significant due to economic and ecological factors [11,12,13,14,15]. The application of the fillers can improve the properties of the sealing element [16,17,18]. 

The use of the fillers for polymer materials constitutes an important area of scientific research at present. The fillers allow for the change of many material properties. That is why, thanks to their application, it is possible to obtain the material properties, which would not be possible to obtain for the reference material [19,20]. The research in this field also comprises composites based on rubber materials. Particularly, it also concerns tribological properties of these kinds of materials [21]. It was also observed that, depending on the type and structure of the filler, the character of material wear changes [22]. 

Research is conducted with the aim of creating materials with better functional characteristics. A lot of various kinds of fillers are studied. The developed new materials find application in various spheres of the industry. The fillers can have different forms. Although, based on the analysis in the literature, one can infer that, in general, carbon-based materials are widely applied as fillers. The fillers introduced into rubber can significantly improve many properties of the material. Even at a relatively low quantity of the applied filler, the mechanical, thermal, and electrical properties of the elastomeric matrix can be improved. Depending on the choice of the type of filler, properties such as tensile strength, elongation at break, and thermal oxidative stability can be improved. They also enable the change of properties such as the friction coefficient and specific wear rate in the case of materials used as the elements, which need to perform relative movement. Various kinds of rubber such as isobutylene-co-isoprene (IIR), polychloroprene (CR) [23], silicone rubber (SR) [24,25,26,27], ethylene-propylene-diene rubber (EPDM) [28,29], petroleum resin, and nitrile rubber (NBR) [30,31,32] are used as a matrix material. In the literature, one can come across broadly discussed research results on the application of carbon-based materials as filler [33,34,35,36]. 

Scientists are also interested in the methods of material optimization, including reinforcement application. The identification and description of the complex structures obtained in this way is a complicated issue [37,38]. 

The research associated with O-ring seals, described in the literature, refers to their reliability. The mechanical behavior of chosen materials, taking into account both static and dynamic strain [38,39,40], is checked during the tests. The influence of the environmental conditions on the change in the material properties is analyzed. Such research shows how the properties were change with the aging test time. On the basis of the studies of the mechanical behavior of rubber, it is possible to study the degradation process of rubber and the material reliability. By comparing the properties of two materials, it is possible to indicate which material is better for use as the sealing element. Materials like NBR and EPDM, which are broadly used materials for sealing rings, were among those tested. From the point of view of the reliability of operation, the processes leading to wear and destruction of the surface of sealing elements are of vital significance. This is because the damage of elements of this kind can lead to faultiness of the whole device [41,42,43,44].

Rubber compounds filled with carbon black, carbon nanotubes, and graphitic filler were among those which were studied. As the research results show, the application of the tested fillers can have a positive influence on the final properties of the products [45,46,47,48,49,50]. In the publications, one can also come across the results and discussions of research on the application of other additions, which will enable the friction force to be lowered during the slide. Fillers, such as the hollow glass beads or micro glass flake fillers, show a similar effect [51,52]. Their application allows the friction force to be reduced and also improves the anti-wear properties of the material. Materials such as styrene-butadiene rubber-based (SBR), EPDM, and NBR were also tested with fillers in the form of fibers [53,54]. As it was ascertained, fillers in this form also have a positive influence on the mechanical and tribological properties. 

As it is clear from the abovementioned review literature, many authors indicate that graphite can be used to improve many properties of polymer materials. The focus of the presented paper was on the comparison of tribological properties of two materials: nitrile rubber (NBR) and fluoroelastomer (FKM). In particular, the influence of the graphite powder application as a filler on friction force for these materials was evaluated. 

## 2. Materials and Methods 

**Figure 1:**
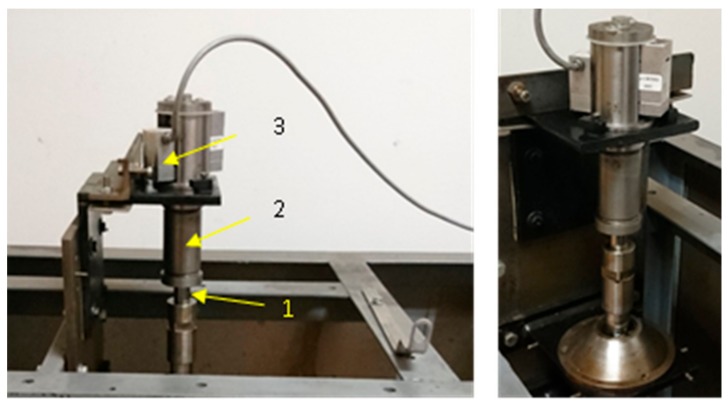
General view of the test stand; 1—rod; 2—chamber; 3—force transducer.

**Figure 2:**
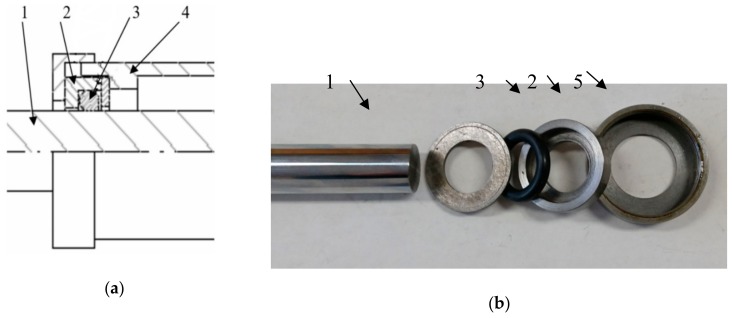
Elements of the test head (**a**) schematic cross-section of the test cell; (**b**) test cell elements; 1—rod; 2—housing; 3—seal; 4—chamber; 5—cover.

With the aim to evaluate the operation of the friction node with the use of the chosen materials, friction force on the O-ring seals was measured. The measurements were made on the original, especially designed stand. The stand enables the measurement of friction force during reciprocation movement. During the assembling of rubber rings in the test head, one can place them in the “groove” with different diameters. The general view of the test stand is shown in Figure 1. However, test elements are depicted in Figure 2. During the test, the surface of the rubber ring that was squeezed in the housing was pressed onto the chromed rod surface. The applied rod was a typical element produced by the Krosno S.A. Poland factory. In accordance with production technologies, the surface of the rod was chromed. The layer thickness was 15 μm, whereas the surface hardness was 57 HRC. 

The shaft surface, which during the tests had contact with the surface of the rubber ring, was characterized by the following roughness parameters: Roughness Average, Average Maximum Height of the Profile, and Root Mean Square Roughness. The results of the surface roughness parameters measurement conducted before and after the tests are presented in Table 1. On the basis of the presented results, it should be assumed that the changes in the rod surface roughness did not take place during the conducted tests. This results from the fact that the test duration was relatively short and the rings were made of a comparatively low-hardness material in comparison to the rod material. The measurements of roughness parameters were performed by Hommel Tester T1000 (Hommelwerke GmbH, VS-Schwenningen, Germany). 

Rubber O-rings were made in the Krakgum Sp. z o.o. factory (Dobczyce, Poland). Two types of rubber, which are commonly used for technical seals like NBR and FKM, were adopted as reference materials. Two series of seals of O-ring type were made from these materials. The first series was made from typical mixtures used during the production of commercial seals, whereas in the second series, the graphite in the form of graphite powder was additionally introduced during mixing of the ingredients. The synthetic graphite powder was defined according to Sigma-Aldrich (Darmstadt, Germany) catalogue no. 28.286-3, with a particle size of 1–2 µm in the amount of 4 weight%.

The basic properties of ring materials are shown in Table 2. The measurements were made according to Polish technical standards: PN-80/C-04238 for hardness and PN ISO 37 for tensile strength and elongation at break. 

The nominal external diameter of the rings was 28 mm, whereas the inner ring’s diameter was 18 mm. For the rings used during the measurements, “houses” of different groove diameters were prepared, in which the rings were placed during the tests; the diameters of particular grooves were chosen according to the experimental designs, so that it would be possible to obtain the assumed difference between the external diameter of the rubber ring and internal diameter of the “house”. The differences between these diameters constituted the input parameter and these are given in Table 3. 

The measurements of friction force during one rod stroke for two changeable parameters, which determine the conditions of cooperation of friction elements, were conducted on the stand. The first one was the maximum velocity of the slide during one stroke, and the second one was the compression of the rubber ring in the housing, i.e., the difference between the external diameter of the ring and the internal diameter of the housing. 

The temperature of the rod surface was measured by the pyrometer Optris GmbH (Berlin, Germany). Each measurement began at a temperature of 25 °C. Since the whole stroke of the rod amounted to 34 mm, a significant change of temperature was not observed during the measurement of friction force. All the measurements were made at temperatures ranging from 25 to 27 °C. 

The force transducer HBM GmbH (Darmstadt, Germany) type S2 was used to measure friction force. The obtained friction forces were compared at different compressions of the ring, as shown in the figures. The measurements were made according to the assumed plan.

Each measurement started at the beginning of the rod stroke. During its stroke, the speed of movement increased to the maximum speed assumed for a given test and then the speed of the slide decreased until the rod came to a standstill. Maximum values of friction force obtained during the stroke were chosen for analyses.

The issues associated with the methods of conducting the research and experimental designs can be found in the literature, for example, in the book by Polański [55]. Statistical Hartley’s designs PS/DS-P were used to realize the measurements. Two input parameters were taken into account in the design. As was mentioned, the input parameters were the velocity of the slide and the compression of the rubber ring in housing. Particular measurement points were chosen based on the designs of the experiment. Controlling factors coded as *x* correspond to minimum, central, and maximum values from the range of real values. In this way, their normalization is described by the following relations.

Central point of Hartley’s designs:

(1) $$x_{ko}=\frac{x_{k\max}+x_{k\min}}{2},$$

and unit of variability:

(2) $$\Delta x_{k}=\frac{x_{k\max}-x_{ko}}{\alpha },$$

where *x*_*k*max_ and *x*_*k*min_ represent the maximum and minimum value of the range of input parameters, respectively, and α represents the so-called “star point”.

The coded value is associated with real values:

(3) $${\hat{x}}_{k}=\frac{x_{k}-x_{ko}}{\Delta x_{k}}.$$

In accordance with the above, the experimental designs included 7 points. All measurements were repeated three times. Real values of the slide speed and ring compression were assumed according to the experimental designs and are provided in Table 3.

The obtained measurement results were approximated. A second-degree polynomial function was assumed as a model. Therefore, the function equation with two variables *x*_1_ and *x*_2_ can be presented as follows:

(4) $$f=b_{0}+b_{1}x_{1}+b_{2}x_{2}+b_{11}x_{1}^{2}+b_{22}x_{2}^{2}+b_{12}x_{1}x_{2}$$

where: *b*_0_,…, *b*_12_—regression factors.

Regression factors can be obtained as a solution of the equation system:

(5) $$b_{0}+b_{1}x_{10}+b_{2}x_{20}+b_{11}x_{10}^{2}+b_{22}x_{20}^{2}+b_{12}\left(x_{10}x_{20}\right)=f_{02},\;b_{0}+b_{1}x_{12}+b_{2}x_{22}+b_{11}x_{12}^{2}+b_{22}x_{22}^{2}+b_{12}\left(x_{12}x_{22}\right)=f_{12},\;b_{0}+b_{1}x_{13}+b_{2}x_{23}+b_{11}x_{13}^{2}+b_{22}x_{23}^{2}+b_{12}\left(x_{13}x_{23}\right)=f_{22},\;b_{0}+b_{1}x_{14}+b_{2}x_{24}+b_{11}x_{14}^{2}+b_{22}x_{24}^{2}+b_{12}\left(x_{14}x_{24}\right)=f_{32},\;b_{0}+b_{1}x_{15}+b_{2}x_{25}+b_{11}x_{15}^{2}+b_{22}x_{25}^{2}+b_{12}\left(x_{15}x_{25}\right)=f_{42},\;b_{0}+b_{1}x_{16}+b_{2}x_{26}+b_{11}x_{16}^{2}+b_{22}x_{26}^{2}+b_{12}\left(x_{16}x_{26}\right)=f_{52},\;b_{0}+b_{1}x_{17}+b_{2}x_{27}+b_{11}x_{17}^{2}+b_{22}x_{27}^{2}+b_{12}\left(x_{17}x_{27}\right)=f_{62}.$$

Based on the obtained solutions, values for the whole range of variability of input parameters *x*_1_ and *x*_2_ were estimated. 

Apart from the conducted measurements on the test stand, the observation of the surface of sealing rings was conducted by using a microscope both before and after the tests in order to compare the studied materials. The pictures of the topography of the surface of the test elements were taken by means of the Joel JSM5510LV (Tokyo, Japan) microscope. 

## 3. Results

Collected measurement data permitted for the presentation of the influence of slide speed and ring compression on friction force for particular materials. The results obtained are presented in the figures. 

**Figure 3:**
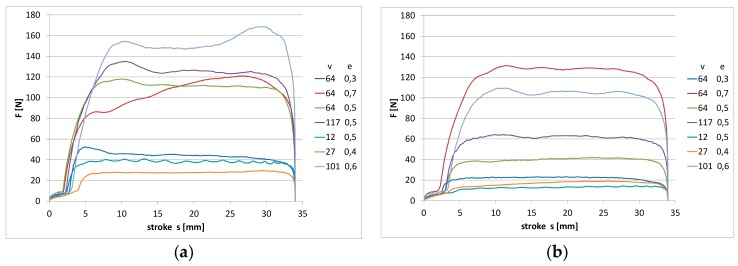
The measured values of friction force during one stroke of the rod: (**a**) nitrile rubber (NBR) pure material; (**b**) NBR material with graphite filler; v {mm/s}; e {mm}.

**Figure 4:**
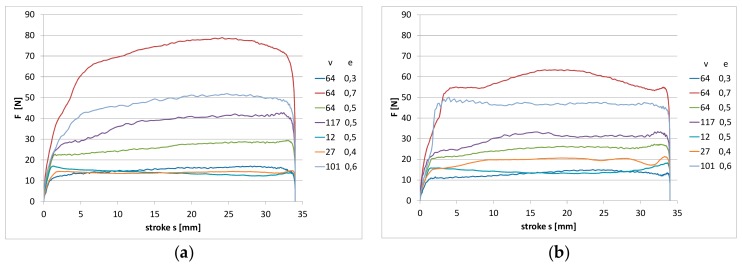
The measured values of friction force during one stroke of the rod: (**a**) fluoroelastomer (FKM) pure material; (**b**) FKM material with graphite filler.

In Figure 3 and Figure 4, measured values of friction force during one stroke of the rod are shown, respectively, for two studied materials. The diagrams were compared for all the tested operating conditions according to the assumed experimental designs. The value of friction force during relative motion of the rubber element and chromed surface of the element on the counterface can achieve very high values. As is visible, both the compression change of the ring and the sliding speed on the rod surface had an influence on the value of friction force. 

In the initial phase of the rod movement for the NBR material (Figure 3), friction force increases at an evidently slower rate. In this part of the stroke of the rod, the deformation of the ring takes place. In the further part of the stroke of the rod, the measured friction force is already the result of the slide of the ring on the rod surface. Since the NBR material was characterized by a smaller hardness (amounting to 71 °Sh), the effect of ring deformation is more visible. As a result of the action of friction force, the ring surface is deformed in the place of contact with the rod surface. In the case of the FKM material, the effect of the deformation is not so clearly visible, as shown in Figure 4. The material had a higher hardness of 79 °Sh, which explains the differences observed in the diagrams. The highest measured values of friction force correspond to the highest values of compression and speed. In the diagram, one can also notice that for some values of speed and compression, fluctuations of friction force occur during the stroke of the rod, which indicates the occurrence of a slip stick phenomenon. During the sliding of the ring on the rod surface, the ring is continuously deformed and the fluctuations of the friction coefficient between the surfaces of the ring and the rod take place. 

In the case of the NBR material, the fluctuations of the value of friction force are most visible at the lowest studied sliding speed (v = 12 mm/s) and the middle range of compression (e = 0.5 mm). The effect was observed for both pure material and after the introduction of graphite powder. However, in case of KFM material (Figure 4), for both pure material and after the introduction of graphite powder, the fluctuations of friction force are visible practically for the whole range of the studied velocities. In the case of base reference NBR, the increase of friction force with the growth of ring compression and the speed of the slide are very clearly visible. Similar observations can be noted for the rubber with the addition of graphite. However, here, lower values of friction force were obtained in the whole range of the change of the studied parameters. 

In Figure 4a, the results obtained for the reference of the FKM material are illustrated. FKM rubber is characterized by a significantly lower friction force in the whole studied range. The registered noted values during the tests were 50% lower than in the case of the NBR material. Moreover, in the case of this material, the application of the graphite addition resulted in a lowering of friction force.

**Figure 5:**
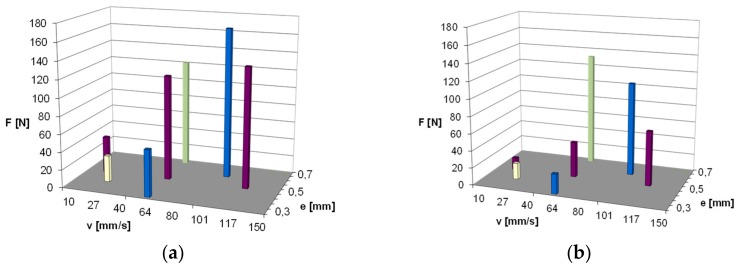
Maximum values of friction force obtained for particular points of the experimental designs: (**a**) NBR pure material; (**b**) NBR material with graphite filler.

**Figure 6:**
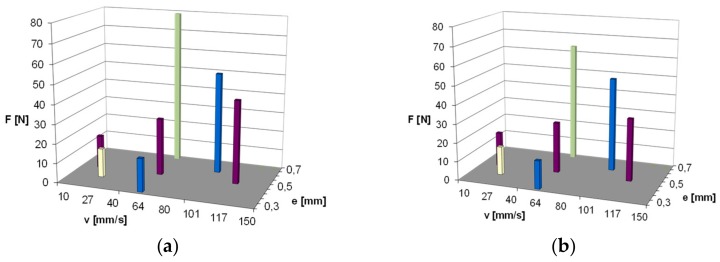
Maximum values of friction force obtained for particular points of the experimental designs: (**a**) FKM pure material; (**b**) FKM material with graphite filler.

For a better depiction of the changes of the values of friction force, maximum values of friction force for particular points of experimental designs obtained during measurement were compared in Figure 5 and Figure 6. As it is visible in the case of the NBR material, the most significant decrease of the maximum values of friction force can be observed for the range of the studied slide speeds from 60 to 120 mm/s, whereby for lower slide speeds (60 mm/s), a significant decrease in the friction force occurred for ring compression e = 0.3 and e = 0.5 mm. However, this effect was no longer observed at the same speed, except for ring compression e = 0.7. For higher speeds, i.e., from 100 to 120 mm/s, the decrease of the maximum value of friction force occurred for both the compressions of 0.5 and 0.6 mm. 

Such observations can be explained by the fact that in the case of the occurrence of graphite particles in the friction zone at higher speeds, the conditions of easier slide are created. Graphite particles cannot remain on the peak of the surface roughness profile at lower speeds and large compression simultaneously. They can solely remain in the valley of the roughness profile at larger pressure, or they are completely removed from the contact surface of the ring and rod. 

As is visible in Figure 6, in general, for all the points of experimental designs, the maximum values of friction force for the FKM material were lower compared to those of the NBR material. Furthermore, in the case of this material, the application of graphite powder gave a noticeable decrease in friction force. However, it is no longer as significant as it was shown to be for the NBR material; although, here, the result of adding graphite powder is also very noticeable for higher slide speeds, i.e., 100 and 120 mm/s. Therefore, the mechanism of action of the added graphite powder can be analogical, as was indicated above. In addition, due to a higher hardness of this material, and a resultant lower deformation, the decrease of the maximum friction force is also visible for the highest values of compression, including the highest compression, e = 0.7, for which the measurement was conducted. 

**Figure 7:**
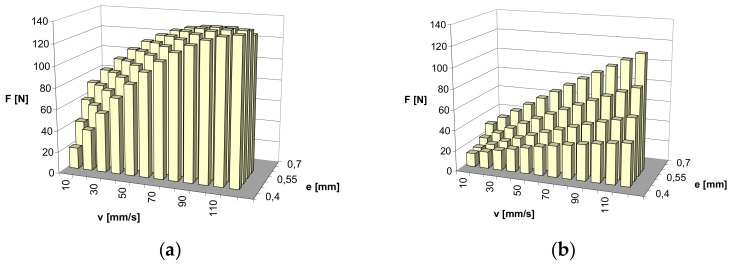
Approximated results of friction force measurements: (**a**) NBR pure material; (**b**) NBR material with graphite filler.

**Figure 8:**
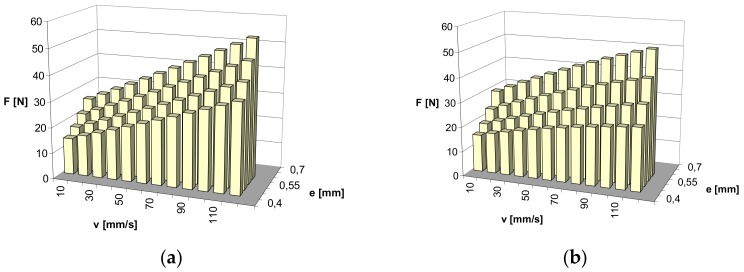
Approximated results of friction force measurements: (**a**) FKM pure material; (**b**) FKM material with graphite filler.

Based on the obtained results of the maximum value of friction force, for all the points of the experimental designs, the values of maximum friction force were estimated for the whole range of the changeability of slide speed v and compression e. A second-degree polynomial was applied for approximation, as previously discussed. The approximated values are presented respectively for the NBR material in Figure 7. Whereas for the FKM material in Figure 8, the dependence of the maximum friction force on slide speed v and compression of the ring e, presented in this way, illustrates the effect of the application of graphite powder as filler.

As is visible in the diagrams, larger changes of friction force depending on the changes of slide speeds and ring compression can be expected for the NBR material. The predicted friction force rapidly increases with the augmented speed of slide and ring compression for pure material without graphite. With the presence of graphite powder, the predicted growth of the friction force is no longer so rapid. However, in general, the expected changes in friction force with the changes in the speed of slide and ring compression are lower for the FKM material. The presence of graphite powder, as in the case of the NBR material, has an influence on the decrease in friction force over the entire range of estimation.

**Figure 9:**
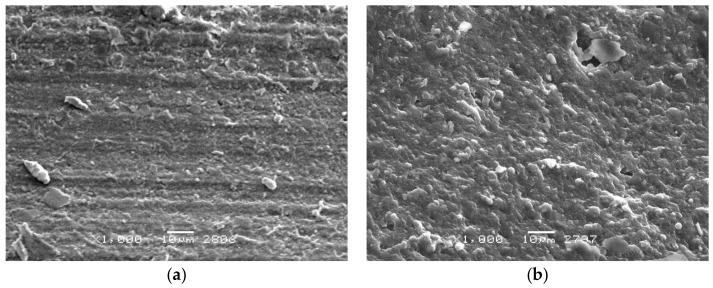
Sample surface—material: NBR (**a**) before the test; (**b**) after the test.

**Figure 10:**
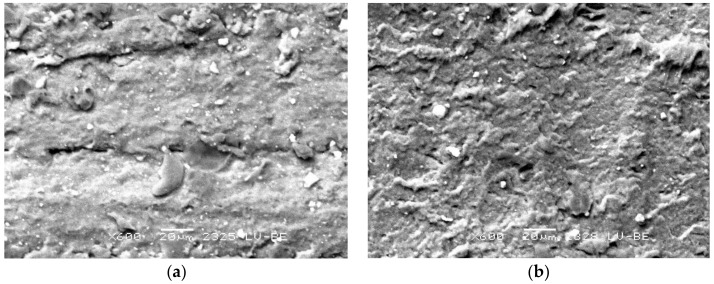
Sample surface—material: FKM (**a**) before the test; (**b**) after the test.

Before conducting the tests and after carrying them out, the ring surfaces were observed. The state of the surface of the new ring made from NBR is shown in Figure 9a. On the surface, the tracks of vulcanization forms are clearly visible. After the tests were carried out, the results of wear were visible on the surface of the rings. The surface of the ring in the area of the wear track was uniform, although, as a result of adhesion of the rubber surface to the rod, substantial elastic deformations occurred, which led to surface undulation and the origin of a characteristic track on the friction surface, as shown in Figure 9b. Analogically, in case of the FKM material, the state of the surface of a new ring is shown before (Figure 10a) and after (Figure 10b) the experiment. The surface within the friction zone is more uniform than in the case of the NBR material. Such a state of the surface indicates that significantly smaller deformations of the ring material occurred during the slide. It is consistent with the registered lower friction force. On the friction track, one can observe uniform wear. It is characteristic for this type of element. The surface of the FKM rubber on the friction track is more uniform in comparison to NBR.

## 4. Discussion

The discussed results of the research illustrate the characteristic of the operation of chosen rubber materials in friction paired with the chromed rod surface. Special cooperation conditions, characterized with the lack of lubrication, were chosen for the analysis. These are very unfavorable conditions. As was observed, they result in very quick changes of the state of the rubber surface elements. The restriction of friction force and wear of this type of elements is significant due to the increase in their durability.

In general, one can observe higher values of friction force at higher values of ring compression and higher speeds of rod movement. Due to the character of the tribological phenomena, the friction force may undergo change even during one stroke of the rod. The application of graphite has an influence on the change of conditions on the contact surface of the ring and rod. Graphite particles can create a kind of lubricating film on the rod surface, which diminishes friction force. As was observed, it is most visible at higher speeds. The graphite can also remain in the valley of the profile of surface roughness. In the case of the material with lower hardness, the decrease in friction force is more visible after the application of graphite powder. For the material of higher hardness, the effect is lower. Such observations can be explained by the accumulation of graphite in the valley of the profile of surface roughness.

As the research has shown, even a slight addition of the graphite can have an influence on friction force. Small particles of graphite can create a relatively permanent layer, which fulfills the role of lubricating film on the surface of the rubber. It can fulfill a particularly significant role in conditions of dry friction. Wang et al. [56] also indicated such a role of graphite in the case of butadiene rubber. In addition, they also indicated its role in the decrease in wear. They also suggested that smaller particles of graphite can be distributed more evenly on the surface and thereby contribute to diminish friction force. 

Considerable differences of the measured values of friction force for the materials of different hardness indicate the significance of the role the strains play in elastomer material. The contact of elastomer with smooth and hard surfaces leads to the origin of strains in elastomer material, which is significant for the friction phenomenon. Persson et al. [57] also indicated the significance of this factor. In the literature, one can find the analyses of the mechanism of the creation of the so-called “third body”, which can be created between two sliding surfaces [58]. These are the structures which are formed on one or two surfaces of the elements, which create a friction pair, usually consisting of degraded polymer particles. During the slide of the elements, such structures can be created and removed from the surface. Graphite powder introduced as filler can play a significant part in creating or modifying such structures. The observed changes in friction force will be the consequence of this. Graphite particles will be able to mix with the products of rubber wear. Graphite powder added to the material of the ring can be found on its surface at the beginning of use of the ring. The material which contains the filler will have the possibility to complete the graphite particles on the contact surface with the rod even during proceeding wear. The observed decrease in friction force after the introduction of graphite can be explained by a course of tribological processes. Due to the number of the factors which decide the friction force, it is very difficult to evaluate it in a theoretical way. In the case of polymers, the conditions of contact between the surfaces of the seal and rod are changing with time. The longer the elements stay in contact without relative movement, the larger the deformation of the surface of the seal can undergo. Friction force depends on the squeeze of the ring. Friction force resulting from the direct contact of the surface of the ring and rod has to be overcome, especially in the initial period of the movement of the elements. This initial period is called start-up friction. As was presented in previous papers, in case of the elements which make relative movement, their tribological characteristic is significant [59]. Elastic deformation of the rubber surface has an influence on the conditions of slide and friction force [60]. That is why the materials, which differed in hardness, were characterized by significantly different values of friction force. As the results of the measurement showed, the differences occurred irrespective of the whether graphite was added or not. The presence of graphite particles in the friction zone has an influence on the change in slide conditions. It seems that it can be clearly explained by the favorable lubricating properties of graphite. In the case of the lack of lubrication, graphite particles may fulfill the role of a solid lubricant. The addition of 4% graphite was applied to the chosen elements in the conducted experiments. The amount was not increased so as not to change vulcanization conditions in comparison to the original materials. 

## 5. Conclusions

The tribological characteristic of two different rubber materials in particular operation conditions was studied in the presented paper. The research in the presented paper focused on the evaluation of the friction-reducing capability of graphite in rubbers of commercial sealing parts. The experimental research presented in this paper led to the following conclusions:

The friction force during relative motion of the rubber element and the chromed surface of the counterface element can achieve very high values. Lower values of the maximum friction force in comparison to the NBR material were noted over the entire range of the conducted measurements for FKM. This confirms that the value of friction force in conditions of a sealing ring operation is influenced by the hardness of the material it is made of. In the case of the two tested materials, the addition of graphite powder had an influence on the decrease of friction force. The particles of graphite, which can be found in the contact zone of the rubber and sealing element, have an influence on slide conditions. 

The obtained results confirm that graphite added as a filler is capable of creating a relatively permanent layer, which has an influence on the decrease of friction force on the surface of the elements of the rubber ring and rod. Graphite particles may fulfill the role of a solid lubricant. We should also pay attention to the fact that a lower friction force is reflected in the character of the wear of the rubber material surface.

## Figures and Tables

**Table 1:** Roughness parameters of rod surface.

	Ra (μm)	Rz (μm)	Rq (μm)
Before test	0.05	0.54	0.07
After test	0.04	0.36	0.06

**Table 2:** The basic properties of ring materials.

Feature	Units	NBR	NBR28	FKM	FKM28
Hardness	°Sh	71	73	79	82
Tensile strength	MPa	13.2	12.6	18.0	18.9
Elongation at break	%	400	340	200	220

**Table 3:** Real values of the variables according to experiment designs.

U	*x*_1_ = v	*x*_2_ = e
1	12	0.5
2	27	0.4
3	64	0.3
4	64	0.5
5	64	0.7
6	101	0.6
7	117	0.5

*x*_1_—real value of rod speed mm/s; *x*_2_—ring compression [mm].
